# Discovery of Nigri/*nox* and Panto/*pox* site-specific recombinase systems facilitates advanced genome engineering

**DOI:** 10.1038/srep30130

**Published:** 2016-07-22

**Authors:** Madina Karimova, Victoria Splith, Janet Karpinski, M. Teresa Pisabarro, Frank Buchholz

**Affiliations:** 1 Medical Systems Biology, UCC, Medical Faculty Carl Gustav Carus, TU Dresden, Germany; 2 Structural Bioinformatics, BIOTEC TU Dresden, Germany; 3 Max Planck Institute for Molecular Cell Biology and Genetics, Dresden, Germany

## Abstract

Precise genome engineering is instrumental for biomedical research and holds great promise for future therapeutic applications. Site-specific recombinases (SSRs) are valuable tools for genome engineering due to their exceptional ability to mediate precise excision, integration and inversion of genomic DNA in living systems. The ever-increasing complexity of genome manipulations and the desire to understand the DNA-binding specificity of these enzymes are driving efforts to identify novel SSR systems with unique properties. Here, we describe two novel tyrosine site-specific recombination systems designated Nigri/*nox* and Panto/pox. Nigri originates from *Vibrio nigripulchritudo* (plasmid VIBNI_pA) and recombines its target site *nox* with high efficiency and high target-site selectivity, without recombining target sites of the well established SSRs Cre, Dre, Vika and VCre. Panto, derived from *Pantoea sp. aB*, is less specific and in addition to its native target site, *pox* also recombines the target site for Dre recombinase, called *rox*. This relaxed specificity allowed the identification of residues that are involved in target site selectivity, thereby advancing our understanding of how SSRs recognize their respective DNA targets.

The precise manipulation of DNA is arguably one of the key technologies that hallmark the rapid progress in biomedical research. Historically, genetic engineering was launched by the discovery of restriction enzymes and DNA ligases allowing to copy and paste desired DNA sequences and thus empirically test the function of DNA sequences (reviewed in)^1^. While revolutionary at the time, conventional DNA modification employing restriction enzymes and ligases encounters limitations due to the abundance of the short recognition sequences of most restriction enzymes. Rare-cutting enzymes, such as meganucleases, circumvent this obstacle because of the longer recognition sites and lower abundance in the genome, but changing their DNA-binding specificity to make them useful for refined genome engineering is challenging^2^. Recently, novel approaches have been successfully developed to expand the variety of DNA-cleaving enzymes, with zinc-finger nucleases (ZFNs), TAL enhancer nucleases (TALENs) or clustered regularly interspaced short palindromic repeat (CRISPR) - associated nuclease (Cas) (CRISPR/Cas9 system) revolutionizing the field of genome engineering (reviewed in)^3^.

Site-specific recombinases (SSRs) constitute a distinctive class of enzymes that possess the unique ability to fulfill both cleavage and immediate resealing of the processed DNA^4^,^5^,^6^. Some SSRs function without cofactors and lead to precise, predictable and efficient genome modifications in animals^7^,^8^ and plants^9^,^10^. In contrast, nucleases induce a double strand break in the genome that has to be repaired by cell intrinsic DNA repair pathways^3^. Hence, the exact DNA sequence after genome editing with nucleases is typically not known.

In particular, tyrosine-type SSRs have found widespread use in biotechnology and biomedical research. Among those, the bacteriophage enzyme Cre and the yeast 2 μ derived Flp recombinase are the most widely used enzymes^6^. Multiple elegant strategies have been developed for the control of the recombination event in a temporal, spatial and cell type-specific manner in animals and plants. For example, the application of SSRs has contributed to overcome difficulties in the investigation of genes required for embryonic development in adult organisms^11^. Furthermore, SSRs are now frequently used in animals to model genetic events underlying many human diseases^12^. The combination of different SSRs in the same cell or organism has made it possible to establish ever increasing sophisticated systems^11^,^13^, and several different enzymes have been recently utilized in synthetic biology to build complex genetic circuits^14^,^15^,^16^. In addition, SSRs are being developed as molecular scissors for genome surgery^17^,^18^,^19^. For these applications, the recombinase target-site specificity has to be altered to recombine a predefined target. For a more guided approach and to accelerate the directed evolution process, it is desirable to understand how these enzymes bind and recombine their targets in a specific manner^20^.

These examples explain the growing interest in the identification of new naturally occurring SSR systems. The large amount of data obtained from sequencing environmental samples^21^, including bacteriophages, provides a convenient starting point to look for new possible SSRs and their recognition targets. Recently, three new SSRs with Cre-like properties have been described that specifically recombine their target sites and can therefore be used in conjunction with the already well-established SSRs^22^,^23^. VCre and SCre encoded on the phage-plasmids p0908 of *Vibrio sp.* and p1 of *Shewanella sp.*, respectively, recombine their 34-bp long target sites, *VloxP* and *SloxP* with high specificity^22^. Likewise, the recently identified Vika recombinase, isolated from the gram-negative bacterium *V. coralliilyticus* recombines its 34-bp target site, vox, with high efficiency and specificity^23^. These enzymes expand the repertoire of available enzymes that are useful for advanced genome engineering and detailed characterization of these enzymes provides guidelines for their applied use. The identification of these, in combination with the here described novel SSR systems Nigri/*nox* and Panto/*pox* will allow the design of more sophisticated genome engineering experiments and advance our understanding on how these enzymes bind their DNA substrates.

## Results

### Identification of two new site-specific recombinase systems: Nigri/*nox* and Panto/*pox*

Recent advances in sequencing technology have dramatically increased our knowledge about the genomes of living matter and thereby provide an enormous resource to perform metagenomic analyses^24^,^25^. These studies deliver a catalogue of genes and proteins isolated directly from living or dead cells found in nature. Comparative genomics can be utilized as a valuable tool to quickly obtain a better understanding of both genes and genomes^26^. Tyrosine recombinases are frequently found in bacteria and their phages. Over 1,300 of these enzymes have been identified and classified through metagenomic analyses to date^27^. However, only a handful of these enzymes have been molecularly characterized in detail and even fewer have been developed for use in applied settings. A main reason for this is that knowing the sequence of the recombinase is not enough to use it. The enzymes act on their DNA target-sites and the identification of these target-sites is typically not straightforward.

To expedite the identification of Cre-like recombinase systems that require no additional cofactors for catalysis, we focused on bacteriophage and plasmid DNA sequences deposited in the ACLAME database and the NCBI nucleotide collection. ACLAME is a database dedicated to a collection and classification of mobile genetic elements, including phage genomes, plasmids and transposons^28^. We utilized the PSI-BLAST algorithm^29^ to reveal a list of candidate genes of previously non-characterized SSRs. In our earlier attempts to identify novel SSRs, we had observed cases of genomic rearrangements either due to viral integration into the bacterial genome or transposon rearrangements. These alterations led to the displacement of the recombinase gene from its native target site and complicated the bioinformatic prediction of the native target site. In order to exclude rearranged hits, we performed a careful analysis of the genetic context of the putative recombinase protein for the presence of hallmark gene clusters present in the P1 phage genome, the host of the Cre/loxP system^30^. Several putative Cre-like proteins were identified with this approach (Supplementary Table 1). Interestingly, Dre recombinase and its target sequence, rox, originally described in phage D6^31^, were identified in our search in two *E. coli* strains (*Escherichia coli* H252 and *Escherichia coli* DEC12A with one point mutation in the latter), indicating that this approach can identify Cre-like SSRs (Supplementary Table 1).

The search for recombination target sites of identified candidate recombinases was performed with the SeLOX search algorithm^32^. We selected a 10-kb region spanning the candidate recombinase genes in order to search for loxP-like sequences. The searches concentrated on lox-like sequences of overall loxP architecture, including a 34-bp long sequence of two 13-bp inverted repeats, separated by an 8-bp spacer. Up to two mismatches breaking the symmetry were allowed in the inverted repeats. Predicted recombinase target site sequences were filtered for the intergenic location, positioned proximally to the corresponding putative recombinase gene. This analysis successfully matched each candidate recombinase with one target site. Overall, five candidate systems comprising a recombinase and a target site were identified. Full sequences of the putative genes and predicted targets can be found in Supplementary Figure 1.

**Figure 1:**
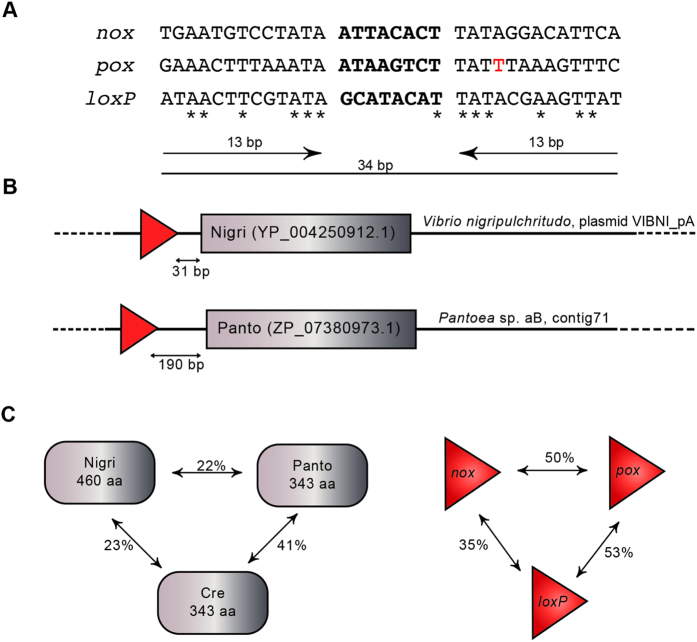
Comparative analysis of Nigri/*nox* and Panto/pox systems. **(A)** Nucleotide sequence alignment of the target sites *nox, pox* and *loxP*. The 13 base pair (bp) inverted repeats are indicated by arrows with the spacer sequences shown in bold. Identical nucleotides are marked with an asterisk. Full symmetry was introduced into the pox site (A to T; marked in red) **(B)** Schematic presentation of the Nigri and Panto coding sequences and the relative position of their native target sites. **(C)** Comparison of the homology of the recombinases and their target sites among each other (in percent). The number of amino acids (aa) for each recombinase is indicated.

Two putative SSR proteins emerged as particularly interesting, because their putative targets showed perfect symmetry or only one asymmetry, respectively (Fig. 1A). Furthermore, both targets were positioned in close proximity to the coding regions of the predicted recombinases (Fig. 1B). We assigned both putative recombinases abbreviations derived from the strains in which they were identified: Nigri, originating from the plasmid VIBNI_pA of *Vibrio nigripulchritudo* and Panto from *Pantoea sp. aB*. Nigri and Panto share low protein identity with Cre (23% and 41%, respectively; Fig. 1C), but conserved residues critical for recombinase catalysis were identified, with all five catalytic residues conserved among tyrosine recombinases being present in both Nigri and Panto (Supplementary Figure 2). Interestingly, Nigri with 460 amino acids is considerably larger than most other Cre-like recombinases. Threading analysis with respect to the Cre 3D-fold identified a large sequence insertion near Nigri’s N-terminus and another one between helices αI and αJ, which apparently do not disturb the overall Nigri/Cre sequence alignment (Supplementary Figure 2). Comparison of the putative target sites *nox* and *pox* to *loxP* revealed that they are dissimilar with only 13 bp out of 34 bp being identical to *loxP* (Fig. 1A). Based on these promising features, Nigri and Panto were chosen for further studies in bacterial and mammalian cell culture assays.

### Nigri and Panto recombine their predicted target sites

**Figure 2:**
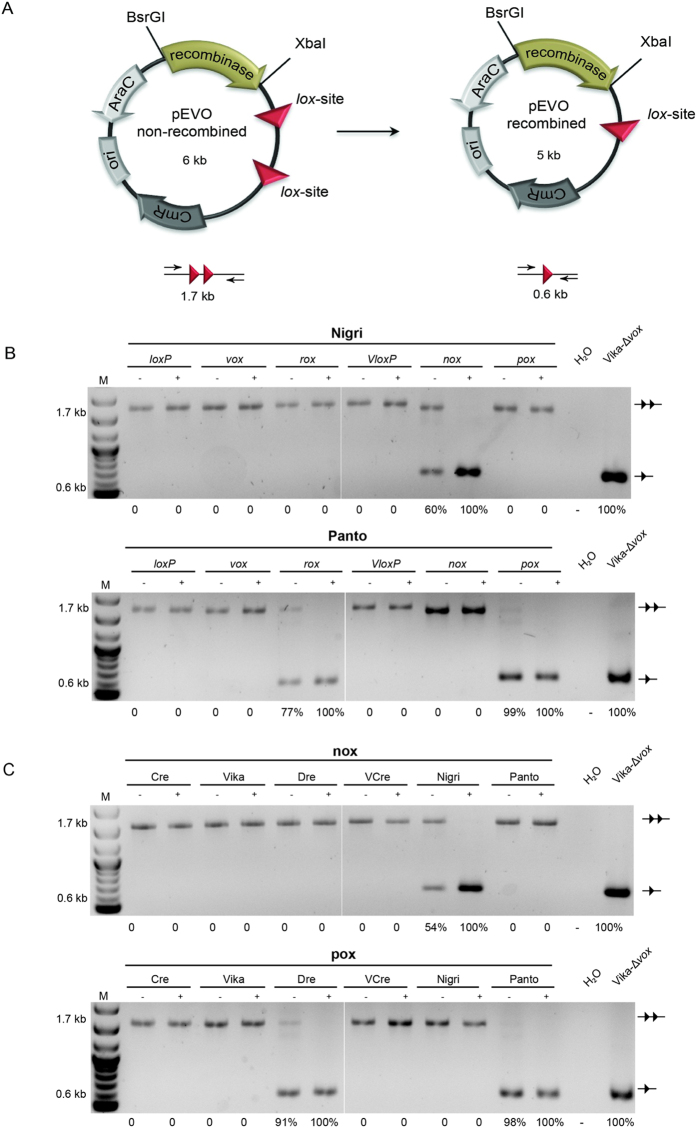
Evaluation of recombination activity of Nigri and Panto. **(A)** Scheme of the recombination assay. Site-specific recombination excises a 1.1-kb DNA fragment, flanked by the recombinase target sites (triangles) from the plasmid. Protein coding genes are depicted by arrows and the origin of replication (ori) is shown as a box. CmR, chloramphenicol resistance gene; AraC, gene encoding the arabinose promoter regulator protein, recombinase, recombinase coding gene. The PCR reaction used to document recombination (0.6 kb) or lack thereof (1.7 kb) is illustrated. **(B**,**C)** Agarose gels showing PCR fragments obtained on pEVO plasmids carrying indicated recombinases and target sites. Addition, or absence of 100 μg/ml L+-arabinose in the growth medium is indicated by “+”, or “−”, respectively. The line with two triangles indicates no recombination, whereas the line with one triangle marks the recombined band. H_2_O shows the negative, water control. The pre-recombined Vika-Δvox vector was loaded as a positive control. M, 1-kb size marker. The calculated percentages of recombination are indicated below each gel picture.

To test whether the predicted target sites for Nigri and Panto are indeed the native substrates for the respective recombinase, we cloned the coding sequence for Nigri and Panto into the pEVO recombination reporter vector^33^, containing either two *nox* or two *pox* target sites, respectively (Fig. 2A). The plasmids were then introduced into *E. coli* and cultured overnight before recombination was investigated. Upon recombinase expression, successful recombination excises a 1.1-kb DNA fragment from the plasmid. This deletion can be detected by PCR with primers flanking the target sites (Fig. 2A). A 1.7-kb fragment and a 0.6-kb fragment, indicative of site-specific recombination, was evident when Nigri was combined with the *nox* target sites and when Panto was combined with the *pox* sites (Fig. 2B). Indeed, both enzymes showed efficient recombination already without the addition of L-arabinose to the medium (approx. 50% recombination for Nigri and about 80% recombination for Panto), indicating that both enzymes are active when expressed at very low levels. Exclusively the 0.6-kb fragment was observed when recombinase expression was induced with 100 μg/ml L-arabinose, demonstrating that both enzymes fully recombine their targets when expressed at high levels. We conclude that Nigri and Panto are Cre-like enzymes and that the predicted DNA sequences are indeed the native recognition targets for each of the two recombinases.

We next tested if Nigri and Panto recombine targets of other well-characterized SSR systems. To investigate this, we cloned Nigri and Panto coding sequences into pEVO plasmids harboring two loxP (Cre), rox (Dre), vox (Vika) or VloxP (VCre) sites, respectively. Plasmids were again transferred to *E. coli* cells and grown with or without the addition of L-arabinose to induce recombinase expression. Nigri did not recombine any of the target sites under non-induced and under induced conditions (Fig. 2B), demonstrating its high target site specificity even when the enzyme is expressed at high levels. In contrast, Panto exhibited relaxed target site specificity as it apparently recombined *rox* – the native target site for Dre recombinase (Fig. 2B) – even under non-induced conditions.

To investigate whether *nox* or *pox* are recombined by other SSRs, we cloned the coding sequences for Panto, Nigri, Cre, Dre, Vika and VCre into pEVO reporter vectors harboring two *nox*, or two *pox* sites, respectively. The *nox* vector was only recombined by Nigri with no recombined bands visible with any of the other recombinases (Fig. 2C). Cre, Vika, VCre and Nigri also did not recombine the *pox* sites in this assay. However, Dre evidently recombined *pox*, even under non-induced conditions (Fig. 2C). Therefore, Dre and Panto are two recombinases with overlapping specificities, both able to recombine *rox* and *pox* sequences.

### Comparative analysis of recombination efficiencies in bacteria

**Figure 3:**
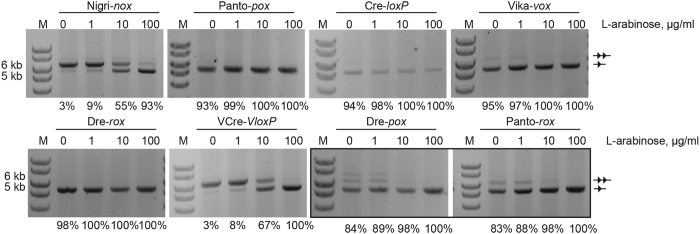
Comparison of recombination efficiency of different recombinase systems. Agarose gels documenting recombination (line with single triangle) of different recombinases. pEVO vectors carrying depicted recombinase – target site pairs were grown under indicated L-arabinose concentrations to induce recombinase expression. Samples are boxed were the cross-recombination activity of Dre on *pox* and Panto on *rox* was assayed. Note that almost full recombination was achieved for Cre on *loxP*, Dre on *rox*, and Panto on *pox* even without addition of L-arabinose to the growth medium. M, 1-kb size marker. The calculated percentages of recombination are indicated below each gel picture.

For the application of SSRs as a molecular tool, it is important to characterize their activities, to compare their recombination efficiencies and specificities and to test the recombinase systems in different assays. To quantify recombination activities at different recombinase expression levels and to allow a side-by-side comparison, we tested Nigri and Panto along with Cre, Dre, Vika and VCre on their target sites in a plasmid-based bacterial assay at different L-arabinose concentrations. In addition, a qualitative comparison of the cross-reactivity of Panto and Dre was performed on *rox* and *pox*, respectively. Plasmids were propagated in *E. coli* for 24 hours and extracted plasmid DNA was assayed for recombination on agarose gels (Fig. 3). This analysis revealed that Panto very efficiently recombines its target site *pox*, but also the *rox* sequence. Even without the addition of L-arabinose to the medium, almost all plasmids had been fully recombined, reflecting that even at very low expression levels the enzyme shows high activity. The activity of Nigri on *nox* was lower and comparable with the activity of VCre on *VloxP*. Therefore, Panto is more active than Nigri on excision substrates in *E. coli*, with Nigri and VCre requiring higher expression level to achieve efficient recombination.

**Figure 4:**
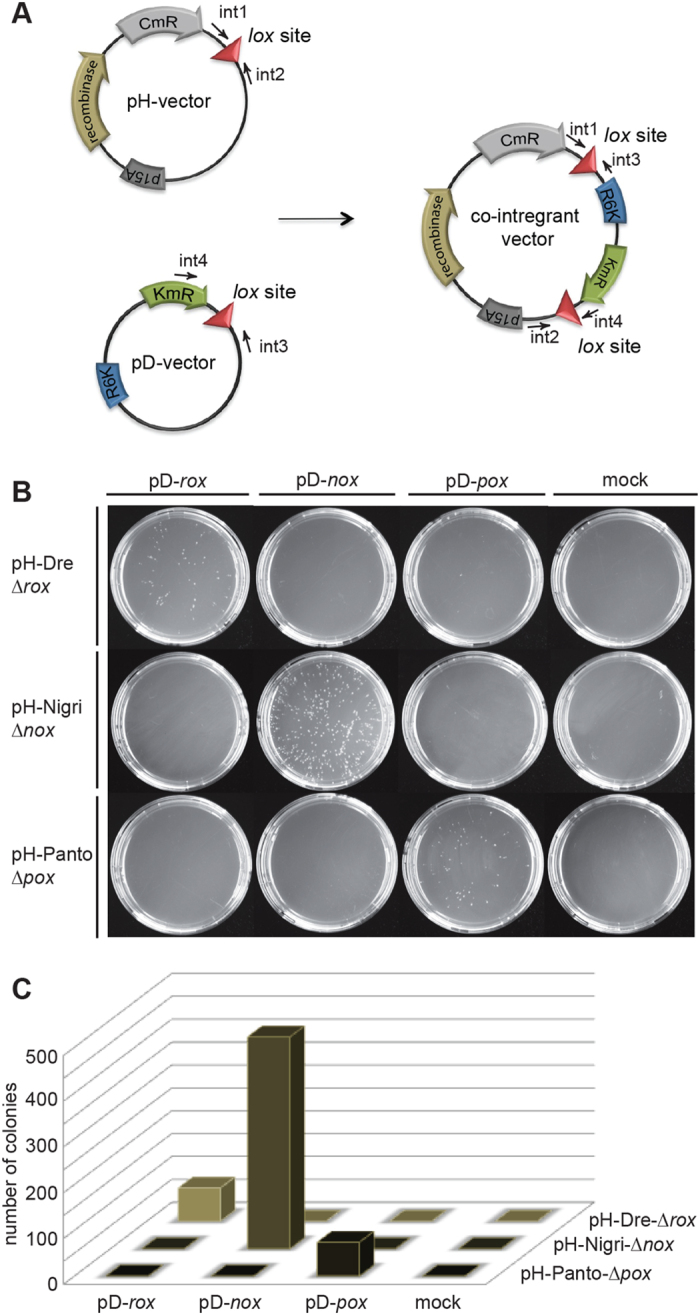
Recombinase co-integration assay. **(A)** Scheme of the plasmid co-integration assay. The host plasmid (pH) carrying the coding sequence for the used recombinases, the chloramphenicol resistance gene (CmR), a p15A origin of replication (p15A) and one respective recombinase target site (triangle) is shown. The donor plasmid (pD) carrying the kanamycin resistance gene (KmR), a R6K origin of replication (R6K) and also one respective recombinase target site (triangle) is depicted. Only combinations of two identical target sites and their corresponding recombinase will produce co-integrant plasmids that survive kanamycin selection in pir-negative cells. Annealing positions for primers used to validate co-integration (int1–4) are shown. **(B)** Colony formation assay of pir-negative *E. coli* cells transformed with indicated plasmid combinations. Colonies only form on site-specific integration of pD and pH plasmids. **(C)** Quantification of the co-integration assay. The number of colonies obtained with indicated pD and pH plasmid combinations are shown.

To test activity of Panto and Nigri in a different experiment, we performed plasmid-integration assays (Fig. 4A). In this assay, recombinase-mediated fusion of the two plasmids, both carrying one recombinase target site and two different antibiotic resistance markers, has to occur for colonies to grow on appropriate culture plates. One of the plasmids (pD-vector) harbored an R6K origin of replication, which requires the pir protein for replication^34^. Only recombinase-mediated co-integration of the pH and pD vectors allows growth of cells on kanamycin-containing medium. To test the integration ability of Nigri and Panto, a panel of host and donor plasmids were generated and co-delivered into *E. coli* cells. Cells were induced by addition of L-arabinose to express the recombinase, and plated on kanamycin and chloramphenicol-containing plates to detect co-integration events. Nigri showed efficient colony formation when provided with constructs containing the native targets on the host and donor plasmids (Fig. 4B). Also Dre and Panto co-integrated rox and pox containg pH and pD vectors, albeit at a lower frequency. In contrast, no colonies were observed when pH vectors where combined with pD vectors carrying a different target sequence and no cross-recombination between rox and pox containing plasmids was observed in this assay, likely because of incompatible spacer sequences of the two target sites. Hence, the recombinases specifically recombined matching donor and recipient plasmids without showing cross-reactivity (Supplementary Figure 3). Surprisingly, and in contrast to the excision assay, the efficiency to co-integrate the two vectors was higher for Nigri/*nox* than for Panto/*pox* (Fig. 4C).

Overall, these results establish Nigri and Panto as two new Cre-like recombinases that perform efficient intra- and inter-molecular recombination without apparent need for additional host co-factors.

### Nigri/*nox* and Panto/*pox* are efficient recombination systems in human cells

**Figure 5:**
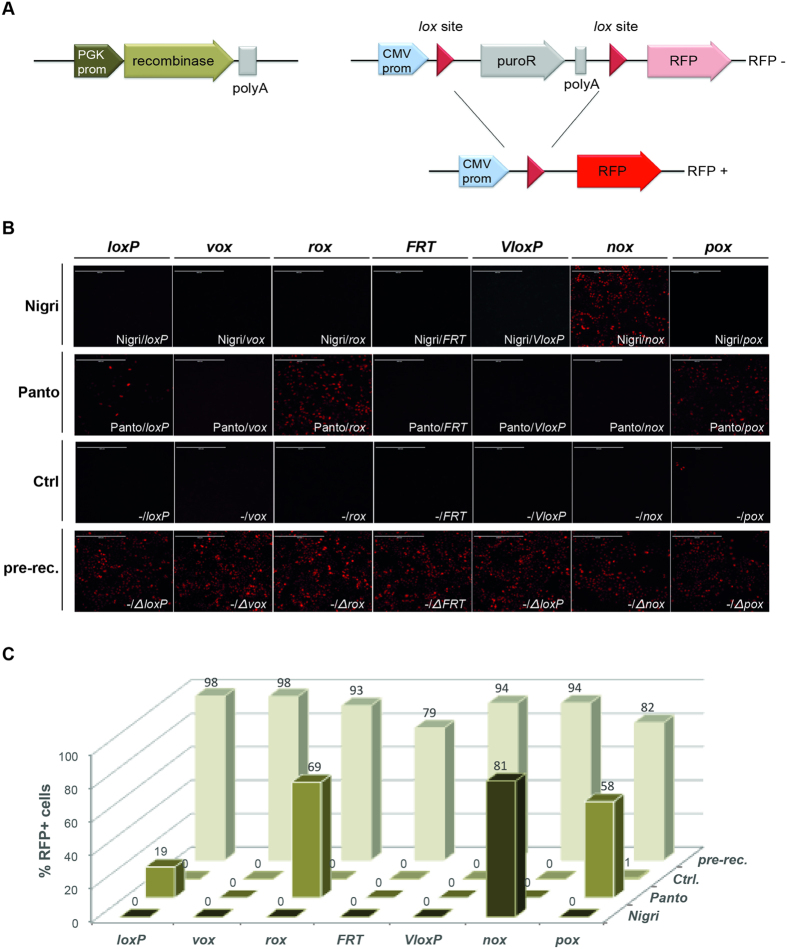
Comparative recombination activity of Nigri and Panto in mammalian cells. (**A**) Scheme of the RFP-based recombination reporter assay. The non-recombined reporter plasmid constitutively expresses the puromycin resistance gene (puroR). On recombination, puroR is removed, resulting in expression of the RFP driven from the cytomegalovirus promoter (CMV prom). Triangles depict lox sites. **(B)** Recombination activity after co-transfection of indicated recombinase expression plasmids and recombination reporter plasmids in HeLa cells. The controls (Ctrl.) show experiments where the recombination reporters were transfected alone. The pre-recombined (pre-rec.) lanes show experiments were the recombined version of the reporter plasmids were transfected alone into HeLa cells. **(C)** Quantification of recombination efficiency. The percentage of RFP positive cells (RFP+) deduced from images shown in (**B**) is presented.

To test the activity and specificity of Nigri/*nox* and Panto/*pox* in mammalian cells, we co-transfected HeLa cells with recombinase expression plasmids and recombination reporter plasmids (Fig. 5A). In the reporter plasmid, a puromycin cassette driven by a CMV promoter is flanked by the different recombinase target sites. Upon site-specific recombination, the puromycin cassette is removed and the CMV promoter now drives expression of red fluorescent protein (RFP). Hence, active recombinases on the respective target sites can be conveniently identified through fluorescence microscopy. When the Nigri expression plasmid was combined with the reporter plasmid carrying *nox* target sites, many cells showed expression of RFP (Fig. 5B,C), showing that Nigri efficiently recombines *nox* sites in mammalian cells. Furthermore, no red fluorescent cells were observed when the Nigri expression plasmid was combined with any other of the tested reporter plasmids, demonstrating the high target-site specificity of this recombinase.

Co-transfection of the Panto expression plasmid with the reporter carrying *pox* sites also produced many RFP positive cells, showing that Panto functions in mammalian cells (Fig. 5B,C). However, as in the *E. coli* assays, Panto also efficiently recombined the reporter plasmid carrying *rox* sites (Fig. 5B). Moreover, a low number of red cells was also observed when the Panto expression plasmid was combined with the *loxP* reporter (Fig. 5B,C). Hence, Panto is an efficient, but a nonspecific recombinase in mammalian cells.

We then tested a panel of established SSRs for their ability to recombine *nox* and *pox* sites. In contrast to Nigri, co-transfection of Cre, Vika, Dre, Flpo, VCre or Panto expression plasmids with the *no*x reporter did not produce RFP-positive cells (Supplementary Figure 4), demonstrating that *no*x sites are not recombined by these recombinases in mammalian cells.

Testing the different recombinases on *pox* sites showed that most enzymes do not recombine this site. However, as in the assays before, we observed that *pox* sites were recombined by more than one recombinase. When the *pox* reporter plasmid was co-transfected with the Panto or the Dre expression plasmid numerous RFP-positive cells became visible (Supplementary Figure 4), further corroborating the cross-reactivity of the Panto/*pox* and the Dre/*rox* systems. Overall, these experiments establish Nigri and Panto as potent enzymes for recombination in mammalian cells.

### Comparison of cytotoxicity of different recombinases

**Figure 6:**
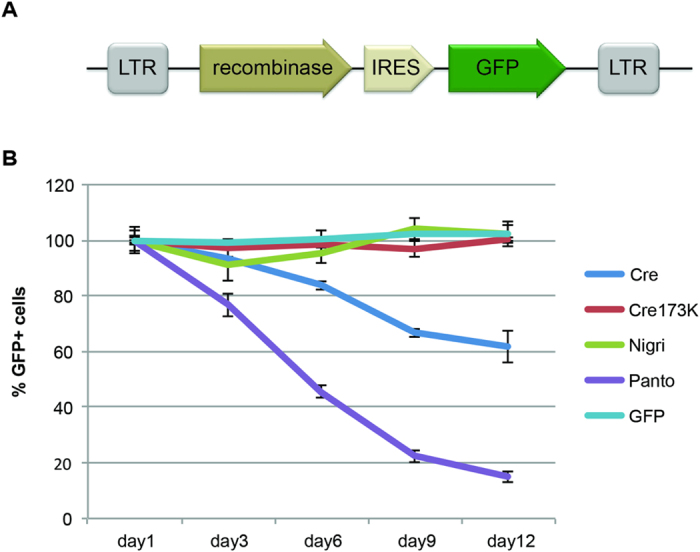
Evaluation of toxicity upon overexpression of recombinases in mammalian cells. **(A)** Schematic representation of the retroviral expression system employed to overexpress indicated recombinases. **(B)** Proliferation effects of overexpression of indicated recombinases in mouse NIH3T3 cells. Cells were infected with bicistronic retroviruses expressing respective recombinases linked to GFP. Every 72 h, cells were analyzed by flow cytometry and the percentage of GFP-positive cells was recorded. Values from the first measurement day were set to 100%. Declining percentages of GFP-positive cells are indicative of a proliferation defect of infected cells. Error bars show standard deviation of the mean, n = 3.

An important aspect for the applied use of SSRs is their specificity towards respective target sites, without acting on any other sequences in the genome of the heterologous host. DNA rearrangements elsewhere in the genome could be deleterious to the cells/organism. Indeed, several studies have reported unwanted side effects when Cre recombinase was highly overexpressed in mammalian cells^35^,^36^,^37^. To test potential toxic effects of Nigri and Panto overexpression in mammalian cells, we constructed retroviral vectors, for co-expression of the recombinase and GFP (Fig. 6A). Viral particles for strong expression of either Cre, an inactive Cre variant^35^, Nigri or Panto were produced. NIH3T3 cells were then infected and the percentage of GFP-positive cells was monitored for 12 days. A reduction in GFP-positive cells over time indicates cytotoxic effects of recombinase overexpression^23^,^35^. Indeed, the number of GFP-positive cells progressively dropped when Cre recombinase was tested in this system, while the catalytically inactive version of Cre had no effect (Fig. 6B). Panto also showed a progressive drop in GFP positive cells, suggesting that strong overexpression of this recombinase is toxic to the cells as well (Fig. 6B). In contrast, the percentage of GFP-positive cells did not change in cells expressing Nigri (Fig. 6B), indicating that overexpression of this recombinases had no negative effect on cell growth/viability. We conclude that high expression of Panto can be toxic in mammalian cells, while high expression of Nigri is well tolerated.

### Relaxed specificity of Panto reveals key residues for target site selectivity

**Figure 7:**
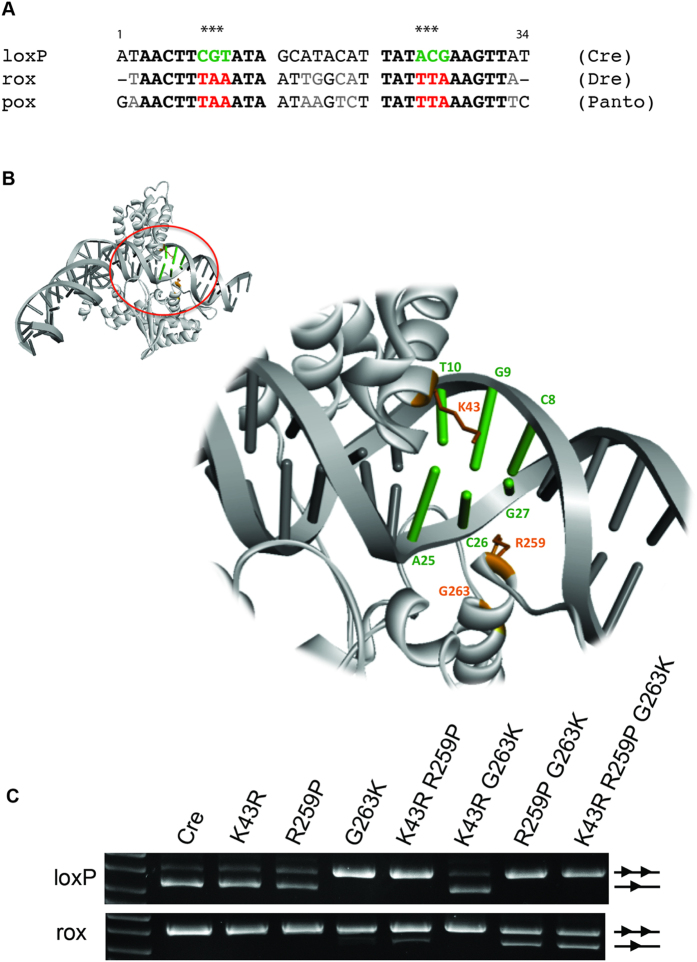
Comparative analysis of Panto, Dre and Cre to identify key residues for recombination specificity in Cre. **(A)** Nucleotide sequence alignment of the target sites *loxP, rox* and *pox*. The three nucleotides that are different in the *loxP* half-site in comparison to *rox* and *pox* are marked with asterisks and highlighted in green and red in loxP and rox/pox, respectively. Nucleotides that differ between *rox* and *pox* are shown in grey. **(B)** Structure of the Cre/*loxP* complex (gray cartoon, PDB ID 1NZB) with a zoom into the region where the three nucleotides changed in rox and pox with respect to *loxP* are located (*i.e.* C_8_G_9_T_10_/A_25_C_26_G_27_ in green). Cre residues facing the DNA in the vicinity of these three changing bases and differing from those in Dre/Panto are displayed in orange (*i.e* K43, R259, G263). **(C)** Agarose gels showing the recombination properties of indicated recombinases on pEVO plasmids carrying *loxP* sites (upper panel), or *rox* sites (lower panel). Lines with one or two triangles indicate recombined and non-recombined bands, respectively.

To investigate the reason for the cross-recombination of Panto and Dre, we compared the sequences of *rox* and *pox* sites. This analysis revealed striking similarities between *pox* and *rox* in the inverted repeat region with only one nucleotide difference (Fig. 7A). Four out of eight positions in the spacer were also identical, making the overall sequence of *pox* and *rox* highly similar. Interestingly, a comparison of Panto and Dre protein sequences revealed only moderate homology between these recombinases (i.e. 60% sequence similarity and 52% sequence identity; Supplementary Figure 2).

We reasoned that a comparative analysis among Cre, Dre and Panto with respect to amino acids known to be relevant for the recognition of loxP by Cre^38^,^39^ might provide new insights into how these enzymes achieve DNA-binding specificity. We hypothesized that residues implicated in Cre DNA recognition being different in Dre and Panto (but identical in these two) might reveal interesting clues about target specificity, and that introducing these residues from Dre/Panto into Cre might change Cre’s DNA binding specificity. We therefore analyzed the 3D structure of Cre in complex with *lox*P (PDBId 1NZB) for protein residues that were different between Cre and Dre/Panto and that faced the DNA major groove near the three bases which differ between *lox*P and *rox/pox* (Fig. 7A). Based on this rationale and analysis, residues K43, R259 and G263 of Cre emerged as particularly interesting candidates for swapping mutations as they face the DNA in the vicinity where *loxP* differs in the base composition to *rox* and *pox* (Fig. 7B). We therefore mutated these residues in Cre to the corresponding amino acid present at positions 43, 259 and 263 in Dre/Panto, that is R, P and K, respectively. In order to see if the mutations K43R, R259P and G263K would affect recombination at Cre’s native *loxP* site, we generated single, double and triple Cre mutants and tested the resulting recombinases on the *loxP* reporter. The mutation K43R and R259P alone had little impact on the recombination efficiency, while G263K, the double mutations (K43R + R259P; R259P + G263K) and the triple mutations (K43R + R259P + G263K) abolished recombination under the conditions tested (Fig. 7C). Interestingly, the K43R mutation rescued the recombination deficiency of the G263K mutation, indicating an integrate relation of these residues.

To investigate whether these mutations would turn Cre into a recombinase with activity on *rox* sites, we tested the same mutants on respective reporter constructs. Indeed, the double mutants with K43R + R259P and R259P + G263K, and the triple mutant K43R + R259P + G263K recombined *rox* sites, with the triple mutant showing the highest recombination activity (Fig. 7C). Hence, the comparative study of related recombinases identified key residues contributing to the site specificity of Cre recombinase.

## Discussion

Metagenomics, the direct genetic analysis of genomes contained within an environmental sample, is a rich source to discover genes encoding new members of protein families. In this study, we used computational methods to predict new Cre-like enzymes and their respective target sites. The obtained results demonstrate that our rational to identifying novel Cre-like enzymes that function without co-factors on their DNA substrates is straight-forward and led to the identification of two new Cre/*loxP*-like SSR systems, Nigri/*nox* and Panto/*pox*.

Nigri recombines its target site *nox* with exquisite efficiency and specificity without cross-recombining with other established SSR systems. Hence, the Nigri/*nox* system should be useful in combination with other SSR systems in sophisticated genome engineering^11^ and synthetic biology^40^ experiments. The Panto/*pox* system might be less suitable in such experiments as it cross-recombines with the Dre/*rox* and possibly the Cre/*loxP* system.

However, the cross-recombination of Panto and Dre can be explored to obtain a better understanding on how these enzymes recognize their targets. By a comparative analysis, we have identified the residues K43, R259 and G263 of Cre to contribute to DNA binding specificity. Interestingly, these residues were also found altered in Cre variants that bind to modified DNA target sequences obtained by directed molecular evolution^17^,^19^, further supporting that these residues play a key role in determining DNA binding specificity. To obtain a detailed molecular understanding about the DNA binding modes of Panto and Dre it would be interesting to obtain 3D atomic models or high resolution structural data (*i.e* by X-ray crystallography) of these proteins bound to their substrates and compare them to already existing SSR/target site structures^6^. This information should help efforts to accelerate the generation of custom specificity enzymes for biotechnology and biomedicine.

An interesting distinction between the Nigri/*nox* and Panto/*pox* systems is their difference in excision versus integrative recombination. While the Panto/*pox* system showed very efficient recombination on excision substrates, even without induction of recombinase expression, the Nigri/*nox* system only efficiently excised the DNA flanked by *nox* sites after induction of the recombinase. This is contrasted by the co-integration assay, in which the Nigri/*nox* system produced about 6 times more colonies. These different properties could have important implications, such as in recombinase mediated cassette exchange (RMCE) experiments, which aim at a high RMCE landing versus excision ratio. It will therefore be interesting to compare the new recombinases in their utility to perform RMCE experiments.

Recently, the release of massive data from large metagenomics projects^41^,^42^ has drastically extended the repertoire of metagenomic sequences. We expect that by harnessing this data, additional Cre-like SSRs and their target sequences will be uncovered in the future. Analysis of metagenomic data has also led to the discovery of a new class of tyrosine recombinases in diverse Polinton-like viruses^43^. None of these SSRs has been molecularly characterized, yet. It would be interesting to uncover the target sites of these enzymes and investigate their recombination properties to test their potential utility in applied genome engineering.

## Methods

### Identification of Nigri/Panto recombinase and *nox*/*pox* target sites

The search for potential Cre-like SSRs was performed using the position-specific iterated Basic Local Alignment Search Tool algorithm [PSI-BLAST/National Center for Biotechnology Information (NCBI)] on the non-redundant sequences in the public DNA database of the NCBI (http://blast.ncbi.nlm.nih.gov/) and BLAST algorithm in ACLAME database^28^. Nigri (YP_004250912.1) and Panto (ZP_07380973.1) were identified from *Vibrio nigripulchritudo* and *Pantoea sp*. aB ctg00071, respectively. The respective target sites *nox* and *pox* were predicted using the Search for Lox-like sequences (SeLOX) algorithm^32^.

### Construction of recombination reporter plasmids

For expression in *E. coli*, codon-optimized DNA sequences for Nigri and Panto were synthesized (Life Technologies Corporation, Grand Island, NY, USA) and cloned into the pEVO vector^33^ via BsrGI and XbaI (NEB, Ipswich, MA, USA) restriction sites. Recombination target sites were introduced into the pEVO plasmids using polymerase chain reaction (PCR). Oligonucleotides (biomers.net GmbH, Ulm, Germany) that contain either of the target sequences *loxP, vox, rox, VloxP, nox* or *pox* (see Supplementary Table 2) were used for amplification of the 1.2 kb kanamycin resistance (KmR) gene. PCR products were purified utilizing QIAquick PCR purification kit (Qiagen, Hilden, Germany), treated with restriction enzymes BglII, XhoI and DpnI (NEB, Ipswich, MA, USA) and used for cloning into pEVO vectors, containing respective recombinases. In order to investigate possible cross-recombination of Nigri and Panto as well as to compare recombination efficiencies, various pEVO constructs carrying different combinations of recombinase gene and a pair of target sites were constructed, accordingly.

For expression of the recombinases in mammalian cells, the coding gene sequences were optimized for expression in human cells (Supplementary Figure 5) and commercially synthesized (Life Technologies Corporation, Grand Island, NY, USA). Fragments were flanked with BsrGI and XbaI restriction sites and a nuclear localization signal was added. The recombinase genes were cloned into pNPK-plasmids as previously described^23^. Sequence information and details of plasmid construction are available on request.

The RFP-based recombination reporter plasmids (pRed) were constructed in a way that site-specific excision of a stop-cassette flanked by co-oriented recombination target sites activates the expression of red fluorescent protein (RFP). The puromycin-resistance gene was amplified from pIRESpuro3-mRedNLS using primers that contain a recombination target site each as well as AgeI restriction sites. The resulting PCR products were digested with AgeI-HF (NEB, Ipswich, MA, USA) and ligated into the pRed plasmid to be located between a SV40 promoter and the RFP gene.

The viral recombinase expression constructs used in the cytotoxicity studies were based on pBabe-puro (Addgene plasmid 1764) and modified to contain an internal ribosomal entry site and a GFP reporter gene as previously described^35^. Nigri and Panto genes were cloned into pBabe-puro vectors using EcoRI and XhoI restriction sites.

### Recombination reporter assays

Functionality of the recombinases Nigri and Panto on their predicted target sites was tested utilizing the pEVO plasmid assay in *E. coli*^33^. Recombination target sites were cloned in co-directed orientation flanking a kanamycin resistance cassette, which hence is excised upon expression of the recombinase. Expression of the recombinases from the pBAD promoter was induced with L-(+)- arabinose (Sigma-Aldrich Chemie GmbH). Single clones containing pEVO plasmid with the recombinase and recombination target sites were cultured overnight in 3 ml LB medium with 25 μg/ml Cm and 100 μg/ml L-(+)-arabinose at 37 °C and 200 rpm. The recombinase-mediated excision event was detected by PCR using primers Int1+ Int2 (for primer sequences, see Supplementary Table 2). Without recombination the PCR product size is 1.7 kb, whereas after recombination the size is 0.6 kb.

To compare the recombination efficiency of the tyrosine recombinases Cre, Vika, Dre, VCre, Nigri and Panto on their native target sites *loxP, vox, rox, VloxP, nox* and *pox*, respectively, recombinase expression was induced with increasing concentrations of L-(+)-arabinose (0, 1, 10 or 100 μg/ml medium) overnight in 3 ml culture volume. Plasmid DNA was isolated using the QIAprep Spin Miniprep Kit (Qiagen, Hilden, Germany) and recombination efficiency was evaluated by agarose gel electrophoresis after digestion with BsrGI (NEB, Ipswich, MA, USA). Quantification of recombination efficiency was performed by measuring the band intensities using ImageJ software applying the Gel analysis feature.

The plasmid co-integration assay was performed as described previously^23^, with plasmids carrying the respective target sites and recombinase genes.

To generate point mutations in the Cre coding sequence, site-directed mutagenesis was performed using the Q5 Site-Directed Mutagenesis Kit (NEB, Ipswich, MA, USA) following the manufacture’s instructions. pEVO plasmids carrying *loxP*, or *rox* sites and the respective recombinase mutants were grown without (assay on *loxP*), or in the presence of 1 mg/ml L-arabinose (assay on *rox*), respectively.

For the mammalian recombination reporter assay, HeLa cells were plated at a density of 4 × 10^4^ cells per well in 24-well dishes and cultured in 4.5 mg/ml glucose Dulbecco’s Modified Eagle’s Medium (DMEM, Gibco^®^-Invitrogen), supplemented with 10% fetal bovine serum (Invitrogen), 100 U/ml penicillin and 100 mg/ml streptomycin (Gibco^®^-Invitrogen). At a confluency of 80–90%, cells were co-transfected with combinations of pNPK plasmid expressing a recombinase and pRed reporters using Lipofectamine^®^ 2000 Transfection Reagent (Invitrogen) according to manufacturer´s instructions. Per well 0.4 μg of DNA (0.2 μg of each plasmid) and 1 μl of Lipofectamine^®^ 2000 reagent diluted in 50 μl Opti-MEM^®^ Reduced Serum Media each were used. 4 h post transfection media was changed and cells further cultured at 37 °C and 5% CO_2_. Cells were imaged 48 h after transfection with an IX81 microscope (Olympus) and examined for nuclear RFP expression.

### Cytotoxicity studies

For the viral delivery, recombinase genes were cloned into pBabe-puro vectors^35^. Retrovirus was produced in Phoenix-GP cells (provided by G. Nolan, Stanford University, Stanford, CA, USA). Phoenix cells were passaged into T75 cell culture flasks in high glucose DMEM (Gibco^®^-Invitrogen). At around 70–80% confluency they were transfected with 18 μg of the pBabe-puro construct, 5 μg p522 plasmid encoding ecotropic envelope and 10 μg pR690 plasmid encoding gag-pol proteins, using Lipofectamine^®^ 2000 Transfection Reagent (Invitrogen). High glucose DMEM was changed after 4 h and transfected cells were cultured overnight at 37 °C. 24 h after transfection media was changed to low glucose DMEM/FBS (Gibco^®^-Invitrogen) and flasks were incubated at 32 °C. 36 h after transfection the first viral supernatant was harvested from the culture, sterile filtrated and frozen at −80 °C.

For transduction, NIH 3T3 mouse fibroblasts were seeded at a density of 2 × 10^5^ cells per well into 6-well plates and grown at 37 °C and 5% CO_2_. The next day, fibroblasts were transduced with the different retroviruses. 0.01 M HEPES pH 7.25 (Media kitchen BIOTEC) and 4 μg/ml Polybrene were added to the thawed retroviral supernatant. Old media was removed from the wells and 1 ml of fresh high glucose DMEM media as well as 1 ml of the supernatant was added onto the cells. The plates were spin-occulated for 30 min at 2500 rpm and 37 °C. After subsequent 4 h incubation at 37 °C, media was changed. The percentage of GFP expressing cells from at least 10,000 cells was tracked over time using a BD FACSCalibur Flow Cytometer (Becton Dickinson Biosciences, San Jose, CA, USA) employing the CellQuest Pro software.

### Computational methods

For the alignment of target site nucleotide sequences, the Clustal Omega Multiple Sequence Alignment tool of the EMBL-EBI was used^44^, and their sequence identity was calculated by dividing the number of identical amino acids (aa)/nucleotides by the total number of aa/nucleotides.

The structure-based sequence alignment of the recombinase proteins was obtained by threading (ProHit, ProCeryon Biosciences^45^,^46^ with the X-ray crystal structure of the Cre/loxP complex as template (PDBId 1NZB). Sequence identity and similarity percentages were calculated with SMS (www.bioinformatics.org).

## Additional Information

**How to cite this article**: Karimova, M. *et al*. Discovery of Nigri/*nox* and Panto/*pox* site-specific recombinase systems facilitates advanced genome engineering. *Sci. Rep.* **6**, 30130; doi: 10.1038/srep30130 (2016).

## Supplementary Material

Supplementary Information

## References

[b1] Emery A. E. Recombinant DNA technology. Lancet 2, 1406–1409 (1981). 6118771 10.1016/s0140-6736(81)92814-2

[b2] Silva G. . Meganucleases and other tools for targeted genome engineering: perspectives and challenges for gene therapy. Curr Gene Ther 11, 11–27 (2011). 21182466 10.2174/156652311794520111 PMC3267165

[b3] Gaj T., Gersbach C. A. & Barbas C. F. 3rd. ZFN, TALEN, and CRISPR/Cas-based methods for genome engineering. Trends Biotechnol 31, 397–405, doi: 10.1016/j.tibtech.2013.04.004 (2013). 23664777 PMC3694601

[b4] Buchholz F. Engineering DNA processing enzymes for the postgenomic era. Curr Opin Biotechnol 20, 383–389, doi: 10.1016/j.copbio.2009.07.005 (2009). 19700301

[b5] Turan S. & Bode J. Site-specific recombinases: from tag-and-target- to tag-and-exchange-based genomic modifications. FASEB J 25, 4088–4107, doi: 10.1096/fj.11-186940 (2011). 21891781

[b6] Van Duyne G. D. Cre Recombinase. Microbiol Spectr 3, MDNA3-0014-2014, doi: 10.1128/microbiolspec.MDNA3-0014-2014 (2015). 10.1128/microbiolspec.MDNA3-0014-2014 26104563

[b7] Heffner C. S. . Supporting conditional mouse mutagenesis with a comprehensive cre characterization resource. Nat Commun 3, 1218, doi: 10.1038/ncomms2186 (2012). 23169059 PMC3514490

[b8] Li S. . Dual fluorescent reporter pig for Cre recombination: transgene placement at the ROSA26 locus. PLoS One 9, e102455, doi: 10.1371/journal.pone.0102455 (2014). 25025770 PMC4099177

[b9] Tungsuchat-Huang T. & Maliga P. Visual marker and Agrobacterium-delivered recombinase enable the manipulation of the plastid genome in greenhouse-grown tobacco plants. Plant J 70, 717–725, doi: 10.1111/j.1365-313X.2012.04918.x (2012). 22268515

[b10] Chong-Perez B. . Excision of a selectable marker gene in transgenic banana using a Cre/lox system controlled by an embryo specific promoter. Plant Mol Biol 83, 143–152, doi: 10.1007/s11103-013-0058-8 (2013). 23591693

[b11] Glaser S., Anastassiadis K. & Stewart A. F. Current issues in mouse genome engineering. Nat Genet 37, 1187–1193, doi: 10.1038/ng1668 (2005). 16254565

[b12] Justice M. J., Siracusa L. D. & Stewart A. F. Technical approaches for mouse models of human disease. Dis Model Mech 4, 305–310, doi: 10.1242/dmm.000901 (2011). 21558063 PMC3097452

[b13] Zhang J. . Conditional gene manipulation: Cre-ating a new biological era. J Zhejiang Univ Sci B 13, 511–524, doi: 10.1631/jzus.B1200042 (2012). 22761243 PMC3390709

[b14] Siuti P., Yazbek J. & Lu T. K. Synthetic circuits integrating logic and memory in living cells. Nat Biotechnol 31, 448–452, doi: 10.1038/nbt.2510 (2013). 23396014

[b15] Bonnet J., Yin P., Ortiz M. E., Subsoontorn P. & Endy D. Amplifying genetic logic gates. Science 340, 599–603, doi: 10.1126/science.1232758 (2013). 23539178

[b16] Friedland A. E. . Synthetic gene networks that count. Science 324, 1199–1202, doi: 10.1126/science.1172005 (2009). 19478183 PMC2690711

[b17] Sarkar I., Hauber I., Hauber J. & Buchholz F. HIV-1 proviral DNA excision using an evolved recombinase. Science 316, 1912–1915, doi: 10.1126/science.1141453 (2007). 17600219

[b18] Hauber I. . Highly significant antiviral activity of HIV-1 LTR-specific tre-recombinase in humanized mice. PLoS Pathog 9, e1003587, doi: 10.1371/journal.ppat.1003587 (2013). 24086129 PMC3784474

[b19] Karpinski J. . Directed evolution of a recombinase that excises the provirus of most HIV-1 primary isolates with high specificity. Nat Biotechnol 34, 401–409, doi: 10.1038/nbt.3467 (2016). 26900663

[b20] Abi-Ghanem J. . Engineering of a target site-specific recombinase by a combined evolution- and structure-guided approach. Nucleic Acids Res 41, 2394–2403, doi: 10.1093/nar/gks1308 (2013). 23275541 PMC3575804

[b21] Gimmler A. & Stoeck T. Mining environmental high-throughput sequence data sets to identify divergent amplicon clusters for phylogenetic reconstruction and morphotype visualization. Environ Microbiol Rep 7, 679–686, doi: 10.1111/1758-2229.12307 (2015). 26061246

[b22] Suzuki E. & Nakayama M. VCre/VloxP and SCre/SloxP: new site-specific recombination systems for genome engineering. Nucleic Acids Res 39, e49, doi: 10.1093/nar/gkq1280 (2011). 21288882 PMC3082901

[b23] Karimova M. . Vika/vox, a novel efficient and specific Cre/loxP-like site-specific recombination system. Nucleic Acids Res 41, e37, doi: 10.1093/nar/gks1037 (2013). 23143104 PMC3553980

[b24] Godzik A. Metagenomics and the protein universe. Curr Opin Struct Biol 21, 398–403, doi: 10.1016/j.sbi.2011.03.010 (2011). 21497084 PMC3118404

[b25] Simon C. & Daniel R. Metagenomic analyses: past and future trends. Appl Environ Microbiol 77, 1153–1161, doi: 10.1128/AEM.02345-10 (2011). 21169428 PMC3067235

[b26] Whitehead A. Comparative genomics in ecological physiology: toward a more nuanced understanding of acclimation and adaptation. J Exp Biol 215, 884–891, doi: 10.1242/jeb.058735 (2012). 22357582

[b27] Van Houdt R., Leplae R., Lima-Mendez G., Mergeay M. & Toussaint A. Towards a more accurate annotation of tyrosine-based site-specific recombinases in bacterial genomes. Mob DNA 3, 6, doi: 10.1186/1759-8753-3-6 (2012). 22502997 PMC3414803

[b28] Leplae R., Lima-Mendez G. & Toussaint A. ACLAME: a CLAssification of Mobile genetic Elements, update 2010. Nucleic Acids Res 38, D57–61, doi: 10.1093/nar/gkp938 (2010). 19933762 PMC2808911

[b29] Altschul S. F. . Gapped BLAST and PSI-BLAST: a new generation of protein database search programs. Nucleic Acids Res 25, 3389–3402 (1997). 9254694 10.1093/nar/25.17.3389 PMC146917

[b30] Sternberg N. & Hoess R. The molecular genetics of bacteriophage P1. Annu Rev Genet 17, 123–154, doi: 10.1146/annurev.ge.17.120183.001011 (1983). 6364958

[b31] Sauer B. & McDermott J. DNA recombination with a heterospecific Cre homolog identified from comparison of the pac-c1 regions of P1-related phages. Nucleic Acids Res 32, 6086–6095, doi: 10.1093/nar/gkh941 (2004). 15550568 PMC534624

[b32] Surendranath V., Chusainow J., Hauber J., Buchholz F. & Habermann B. H. SeLOX–a locus of recombination site search tool for the detection and directed evolution of site-specific recombination systems. Nucleic Acids Res 38, W293–W298, doi: 10.1093/nar/gkq523 (2010). 20529878 PMC2896191

[b33] Buchholz F. & Stewart A. F. Alteration of Cre recombinase site specificity by substrate-linked protein evolution. Nat Biotechnol 19, 1047–1052, doi: 10.1038/nbt1101-1047 (2001). 11689850

[b34] Stalker D. M., Filutowicz M. & Helinski D. R. Release of initiation control by a mutational alteration in the R6K pi protein required for plasmid DNA replication. Proc Natl Acad Sci USA 80, 5500–5504 (1983). 6310578 10.1073/pnas.80.18.5500 PMC384285

[b35] Loonstra A. . Growth inhibition and DNA damage induced by Cre recombinase in mammalian cells. Proc Natl Acad Sci USA 98, 9209–9214, doi: 10.1073/pnas.161269798 (2001). 11481484 PMC55399

[b36] Schmidt E. E., Taylor D. S., Prigge J. R., Barnett S. & Capecchi M. R. Illegitimate Cre-dependent chromosome rearrangements in transgenic mouse spermatids. Proc Natl Acad Sci USA 97, 13702–13707, doi: 10.1073/pnas.240471297 (2000). 11087830 PMC17639

[b37] Pugach E. K., Richmond P. A., Azofeifa J. G., Dowell R. D. & Leinwand L. A. Prolonged Cre expression driven by the alpha-myosin heavy chain promoter can be cardiotoxic. J Mol Cell Cardiol 86, 54–61, doi: 10.1016/j.yjmcc.2015.06.019 (2015). 26141530 PMC4558343

[b38] Guo F., Gopaul D. N. & van Duyne G. D. Structure of Cre recombinase complexed with DNA in a site-specific recombination synapse. Nature 389, 40–46, doi: 10.1038/37925 (1997). 9288963

[b39] Ennifar E., Meyer J. E., Buchholz F., Stewart A. F. & Suck D. Crystal structure of a wild-type Cre recombinase-loxP synapse reveals a novel spacer conformation suggesting an alternative mechanism for DNA cleavage activation. Nucleic Acids Res 31, 5449–5460 (2003). 12954782 10.1093/nar/gkg732 PMC203317

[b40] Flintoft L. Synthetic biology: A circuit to remember. Nat Rev Genet 14, 239, doi: 10.1038/nrg3450 (2013). 23438869

[b41] Williamson S. J. . Metagenomic exploration of viruses throughout the Indian Ocean. PLoS One 7, e42047, doi: 10.1371/journal.pone.0042047 (2012). 23082107 PMC3474794

[b42] Sunagawa S. . Ocean plankton. Structure and function of the global ocean microbiome. Science 348, 1261359, doi: 10.1126/science.1261359 (2015). 25999513

[b43] Yutin N., Shevchenko S., Kapitonov V., Krupovic M. & Koonin E. V. A novel group of diverse Polinton-like viruses discovered by metagenome analysis. BMC Biol 13, 95, doi: 10.1186/s12915-015-0207-4 (2015). 26560305 PMC4642659

[b44] Sievers F. . Fast, scalable generation of high-quality protein multiple sequence alignments using Clustal Omega. Mol Syst Biol 7, 539, doi: 10.1038/msb.2011.75 (2011). 21988835 PMC3261699

[b45] Sippl M. J. Boltzmann’s principle, knowledge-based mean fields and protein folding. An approach to the computational determination of protein structures. J Comput Aided Mol Des 7, 473–501 (1993). 8229096 10.1007/BF02337562

[b46] Sippl M. J. & Weitckus S. Detection of native-like models for amino acid sequences of unknown three-dimensional structure in a data base of known protein conformations. Proteins 13, 258–271, doi: 10.1002/prot.340130308 (1992). 1603814

