# Supplementary material for: Discovery of Nigri/nox and Panto/pox site-specific recombinase systems facilitates advanced genome engineering
Source: Sci Rep. 2016 Jul 22;6:30130. doi: 10.1038/srep30130 (PMC4957104; doi:10.1038/srep30130)
Supplement: Supplementary Information [file srep30130-s1.pdf]

Supplementary information

Discovery of Nigri/*nox* and Panto/*pox* site-specific recombinase systems facilitate advanced genome engineering.

Madina Karimova, Victoria Splith, Janet Karpinski, M. Teresa Pisabarro & Frank Buchholz

```
GenBank Accession number
Origin
Amino acid sequence
Putative target site

WP_000543934.1
Salmonella enterica subsp. enterica serovar Newport str. SL254 plasmid pSN254
mhdtnndkee lvshakvnvp aeqsirgell psssslrniq dnpavaylvs lgskrsrqtm
ssflnivakm igfqnlrdca wssmrrhhil avlemlgdag kapatintyl salkgvalea
wtmkqidtds fqhikqvrsv rgsrlpkgra lerheirslf ftcesdssak glrdaailgv
llgcglrrse ivaldmgsmi ykdralkvlg kgnkermayv pggawkrldk wveevrgthe
gplfprirrf ddvtgermsd qaiyhiletr rveaglemfa phdlrrtfas smldngediv
tvkdamghss iattqkydrr gderlkrasq rldiad
ATAATTATGATAATTACCGTATTATCGTAATTAT

YP_004250912.1, Nigri
Vibrio nigripulchritudo plasmid VIBNI_pA
mdkhhllstr ttaptdgsdf nsdnvtknhh ailieralas alarpgllle thfqpiyqra
drlgvdlsdc psffvatqrl laqhhqhgag lsprsleqfh savrvftqwc hvnrrtalpa
sadtfvlfar snaphitlst lhiyvwairk lhlitglvdp tdsqavkqhl srikkqkiaq
fdtaqdqavp lsdedyrtam tllmaddhpi swrdatllgl ayhtmlrqse lvriqlthiq
prsdgdwtle ipytktnktg rseyvtlphy lmpilqryls lcgdrtltdp gylfvpltrs
gvprrqrmhv tstprapsgv tprrktrggi qfapvapdav tdsiaappdp apiseevqpv
apklvartlk kigerlatht sspqadrays ghsarvgrai hlleigarke divkagrwks
dimferytrq ydvndgylaq irqaedrhwh aqteppdstg
TGAATGTCCTATAATTACACTTATAGGACATTCA

ZP_07380973.1, Panto
Pantoea sp. aB ctg00071 contig 71  whole genome shotgun sequence
mkhlaqiktg alpaeltqlt pediannlrr fiadkaayse ntfrdllsvi rrwafwcner
dvgylpidpe lareyflqma esglasstid khyammnmlc resglpdlrg svdlkrsmkr
irreavlqge rtgqavpfrl pdlqllshlm grsdrltdqr nlaflfvayn tlcrmselsr
irvrdldisd sghvvinlsh tktmvtaagv ikhlsraaag hlmhwlelsg lihhpdamvf
gpvrhnntag vsekpmsapa tekifkdawd llgkepvqdn kgryakwsgh sarvgaamdm
aerdatitqi mqegtwqdpk tvmrylrrse sqkgkmsgil dge
GAAACTTTAAATAATAAGTCTTATATAAAGTTTC

ADW7730.1
Rahnella sp. Y902 plasmid pRAHAQ02
mksareipci flridvalsv tgsslfklym gklspinqal paiqaeevvl arlkefvqdk
eafspntwrq lmsvmrichr wsiensrsfl pmlpadlrdy lnwlqesgra sstiathgsl
ismlhrnagl ippntsplvf ravkkinrva vvtgertgqa vpfrledlle ldalwsdsis
lrhkrdlafl hvaystllri selarlrvrd isratdgrii lnvsytktiv qtggliksls
sqssrrltew msvsginaep daflfcpvhr sgsatlsvtr plstpaiesi faqawltiga
gepiipnkgr ytawtghsar vgaaqdmagr gyavaqimqe gtwkkpetlm ryirnlqahe
gamtdimeks tldhnntk
CTAACATTATATAATGGGACTTATATAAAGTTAG


NP_058395.1
Salmonella enterica subsp. enterica serovar Typhi plasmid R27
mnskpvtrqf edsdlhqelv tfevpnndlk elifyfshmk yntaktylqw lrswnewyqa
nagkegneaw passlpvtep pllayldylq gslshssikg clhalnsihr kaldrpgiit
skvksilasl eqaeareqkv trqatpflvs dlkalikahg ttqsvrklrd lciiwtgfet
llrsaelrri rmqdlvlneq tgsftltvyr tkstvstllt yhltphltat lirlmdmvkr
dqqshpkdyl fqavnyqdsg ymppgwglrs kgneintllk nhnmpyrptr ppigkngkpi
ivddegmlsk ntllrafeaf wdelhpqeag trcwtghsvr vggaielana gythlqimem
gnwsnpemvs ryirnidagk kamtkfmrea lde
CTTAATGCTAATGTATGCGTCCATTAGCTTTAAG
```

Supplementary Figure 1: **Putative Cre-like recombinases and their predicted target sites.** Genbank accession numbers, the organism of origin, the amino acid sequence and the putative target sites are shown.

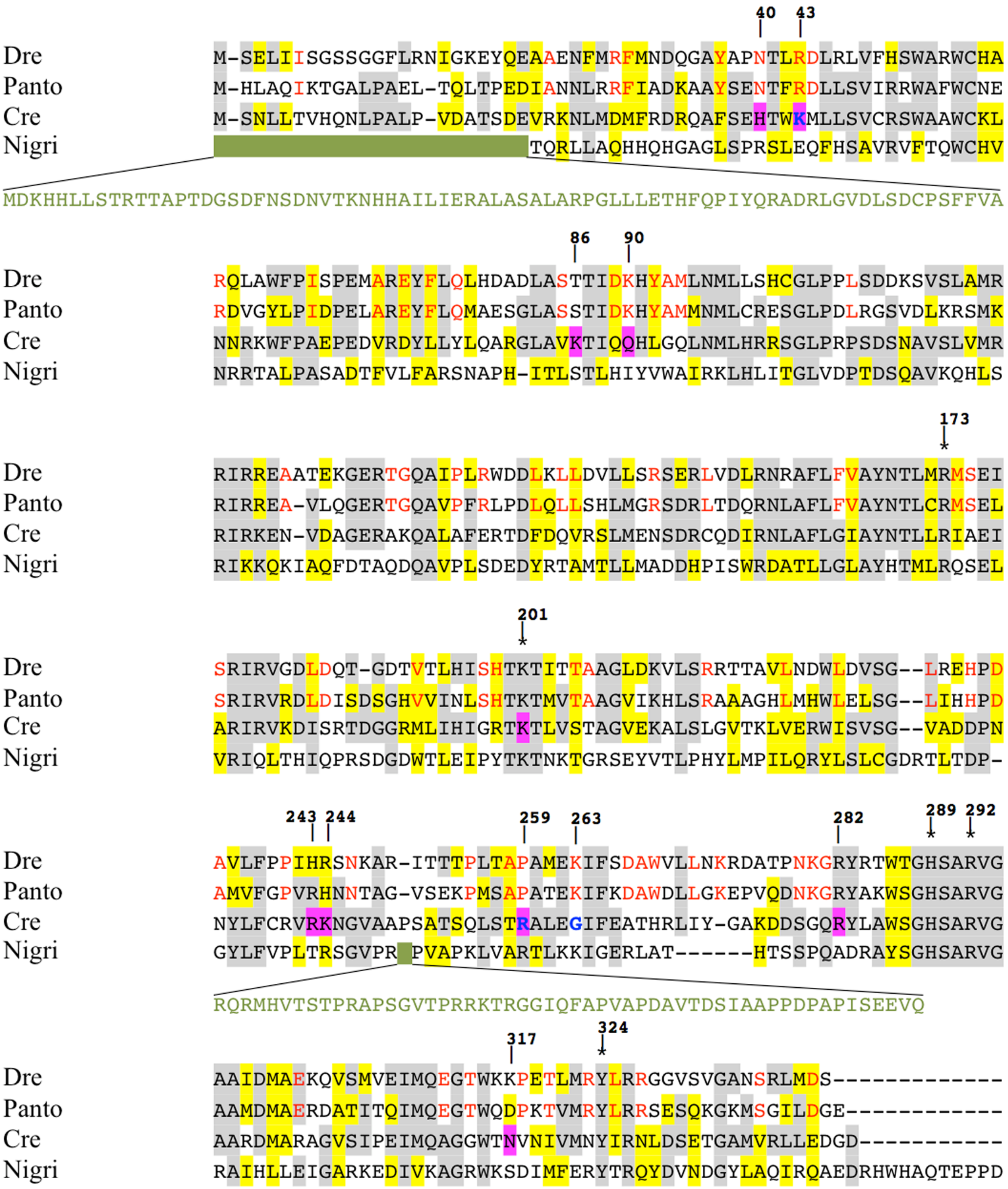

	Dre	Panto	Cre	Nigri
Dre		52	39	15
Panto	60		40	15
Cre	49	50		17
Nigri	23	20	25	

◺ % SIM
◹ % ID

Supplementary Figure 2: **Structure-based sequence alignment of the recombinase proteins Cre, Dre, Panto and Nigri.** The sequences of the recombinase proteins are aligned based on threading (ProHit, ProCeryon Biosciences) and the crystal structure of Cre in complex with *loxP* as template (PDB ID 1NZB). Sequence identity and similarity with respect to Cre are displayed in gray and yellow, respectively. Relevant mentioned residues are numbered, and the ones involved in

catalysis are marked with asterisks. Sequence insertions in Nigri with respect to Cre are represented in green. Pink boxes are indicating residues of Cre contacting *loxP* based in the crystal structure (PDB ID 1NZB) and molecular dynamics simulations by Abi-Gahnem *et al.* [1]. The residues conserved in both Dre and Panto but differing with respect to Cre are shown in red. From those, the ones pointing towards the DNA major groove in the area of three changing bases of *rox/pox* with respect to *loxP* ($C_8G_9T_{10}/A_{25}C_{26}G_{27}$) are shown in bold and blue. Sequence identity (ID) and similarity (SIM) percentages calculated with SMS (www.bioinformatics.org) are displayed at the bottom.

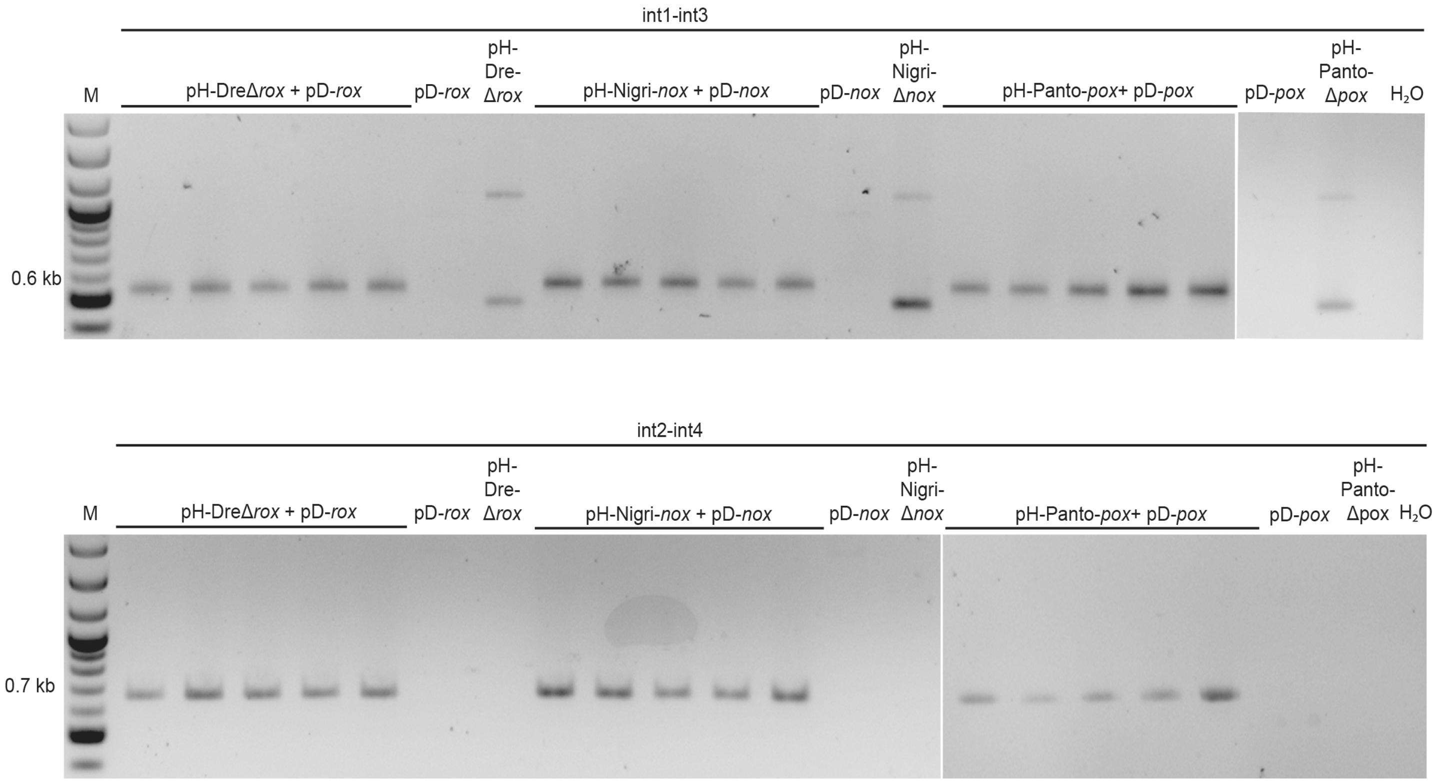

Supplementary Figure 3: **Validation of recombinase mediated co-integration of pH and pD plasmids.** Agarose gels of PCR reactions on DNA isolated from five colonies each obtained with indicated pH and pD combinations with indicated primers (int1-int3, int2-int4) and vectors as depicted in Figure 4 are shown. Controls on the individual pD and pH plasmids and negative water controls (H_2O) are indicated. All examined colonies produced the band sizes expected from a co-integration of pD and pH vectors (0.6 kb and 0.7 kb, respectively). M, 1-kb marker.

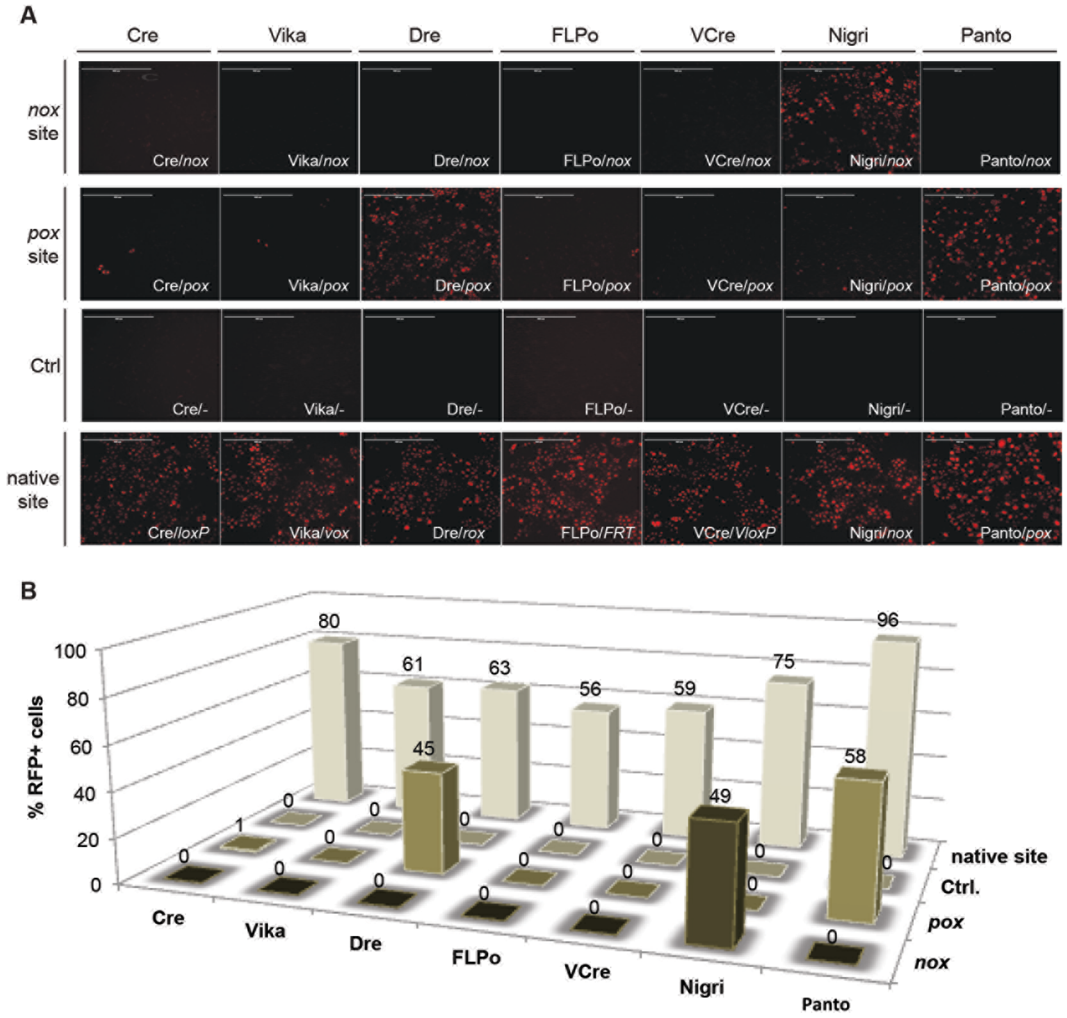

Supplementary Figure 4: **Comparative recombination analysis of selected recombinases on *nox* and *pox* in mammalian cells. (A)** Recombination activity after co-transfection of indicated recombinase expression plasmids and recombination reporter plasmids in HeLa cells is shown. The controls (Ctrl) show experiments where the recombinase expression plasmids were transfected alone. As positive controls the recombinase expression plasmids were co-transfected with recombination reporter plasmids carrying their native sites. **(B)** Quantification of recombination efficiency. The percentage of RFP positive cells (RFP+) deduced from images shown in (A) is presented.

>NLS-Nigri (human_codon_optimized)
ATG**AAGAAAAAGCGGAAGGTG**GACAAGCACCATCTGCTGAGCACCAGAACCACCGCCCCTACCGACGGCAGCGACT
TCAACAGCGACAACGTGACCAAGAACCACCACGCCATCCTGATCGAGAGAGCCCTGGCCTCTGCCCTGGCTAGACCT
GGACTGCTGCTGGAAACCCACTTCCAGCCCATCTACCAGCGGGCCGATAGACTGGGCGTGGACCTGAGCGATTGCC
CCAGCTTCTTTGTGGCCACCCAGAGACTGCTGGCCCAGCACCATCAGCATGGCGCTGGACTGAGCCCCAGAAGCCTG
GAACAGTTCCACTCTGCCGTGCGGGTGTTCACCCAGTGGTGCCACGTGAACAGAAGAACCGCCCTGCCTGCCAGCGC
CGACACCTTCGTGCTGTTCGCCAGAAGCAACGCCCCCCACATCACCCTGAGCACCCTGCACATCTACGTGTGGGCCA
TCAGAAAGCTGCACCTGATCACCGGCCTGGTGGACCCCACAGATAGCCAGGCCGTGAAGCAGCACCTGAGCCGGAT
CAAGAAGCAGAAGATCGCCCAGTTCGACACCGCCCAGGATCAGGCCGTGCCTCTGAGCGACGAGGACTACAGAACC
GCCATGACCCTGCTGATGGCCGACGACCACCCCATCTCTTGGAGAGATGCCACCCTGCTGGGCCTGGCCTACCACAC
CATGCTGAGACAGAGCGAGCTCGTGCGCATCCAGCTGACACACATCCAGCCCAGAAGCGACGGCGACTGGACCCTG
GAAATCCCCTACACCAAGACCAACAAGACCGGCCGCAGCGAGTACGTGACCCTGCCCCACTACCTGATGCCCATCCT
CCAGAGATACCTGAGCCTGTGCGGCGACAGAACCCTGACCGATCCCGGCTACCTGTTCGTGCCACTGACCAGATCCG
GCGTGCCCAGACGGCAGAGAATGCACGTGACCAGCACCCCCAGAGCCCCTAGCGGAGTGACCCCCAGAAGAAAGAC
CAGAGGCGGCATCCAGTTCGCCCCCGTGGCTCCTGATGCCGTGACCGATTCTATTGCCGCCCCTCCCGATCCCGCCC
CTATCTCTGAAGAGGTGCAGCCCGTGGCCCCTAAGCTGGTGGCCAGAACACTGAAGAAGATCGGCGAGCGGCTGGC
CACACACACCAGCTCTCCACAGGCCGACAGAGCCTACAGCGGACACAGTGCCAGAGTGGGCAGAGCCATTCATCTGC
TGGAAATCGGCGCCAGAAAAGAGGACATCGTGAAGGCCGGCAGATGGAAGTCCGACATCATGTTCGAGCGGTACACC
CGGCAGTACGACGTGAACGATGGCTACCTGGCCCAGATCAGACAGGCCGAGGACAGACATTGGCACGCCCAGACCG
AGCCCCCTGATAGCACCGGCTGA

> **NLS**-Panto (human_codon_optimized)
ATG**AAGAAAAAGCGGAAAGTG**AAGCACCTGGCCCAGATCAAGACAGGCGCCCTGCCTGCCGAACTGACCCAGCTGA
CCCCAGAGGACATTGCCAACAACCTGCGGCGGTTTATCGCCGACAAGGCCGCCTACAGCGAGAACACCTTCCGGGAC
CTGCTGAGCGTGATCCGCAGATGGGCCTTCTGGTGCAACGAGCGGGATGTGGGCTACCTGCCCATCGACCCTGAGC
TGGCCCGCGAGTACTTTCTCCAGATGGCCGAGTCTGGCCTGGCCAGCAGCACCATCGATAAGCACTACGCCATGATG
AACATGCTGTGCAGAGAGAGCGGCCTGCCCGACCTGAGAGGCTCCGTGGATCTGAAGCGGAGCATGAAGCGGATCA
GACGCGAGGCTGTGCTCCAGGGCGAGAGAACAGGACAGGCCGTGCCCTTCAGACTGCCCGATCTCCAGCTGCTGAG
CCACCTGATGGGCAGAAGCGACAGACTGACCGACCAGCGGAACCTGGCCTTCCTGTTTGTGGCCTACAACACCCTGT
GCCGGATGAGCGAGCTGAGCCGGATCAGAGTGCGGGACCTGGACATCAGCGATAGCGGCCACGTCGTGATCAACCT
GAGCCACACCAAGACAATGGTCACAGCCGCTGGCGTGATCAAGCACCTGAGCAGAGCCGCCGCTGGACACCTGATG
CACTGGCTGGAACTGTCCGGCCTGATCCACCACCCCGACGCTATGGTGTTTGGCCCCGTGCGGCACAACAACACAGC
CGGCGTGTCCGAGAAGCCCATGTCTGCCCCTGCCACCGAGAAGATTTTCAAGGACGCCTGGGATCTGCTGGGCAAAG
AACCCGTGCAGGATAACAAGGGCAGATACGCCAAGTGGAGCGGCCACAGCGCAAGAGTGGGAGCCGCTATGGATAT
GGCCGAGCGGGACGCCACCATCACCCAGATCATGCAGGAAGGCACCTGGCAGGACCCCAAGACCGTGATGAGATAC
CTGCGGAGAAGCGAGAGCCAGAAAGGCAAGATGAGCGGCATCCTGGACGGCGAGTGA

Supplementary Figure 5: **Nigri and Panto codon optimization.** DNA sequences encoding human codon optimized recombinases Nigri and Panto are shown. Nuclear localization signals (NLS) are highlighted in bold and underlined.

Supplementary Table 1

Protein NCBI ID	Protein size	Protein identity to Vika/Cre/Dre	Strain	Origin	Hit source
WP_000543934.1	336 aa	14% (Vika)<br>19% (Cre)<br>18% (Dre)	Salmonella enterica subsp. enterica serovar Newport str. SL254	plasmid pSN254	ACLAME
YP_004250912.1<br>Nigri	460 aa	27% (Vika)<br>23% (Cre)<br>22% (Dre)	Vibrio nigripulchritudo	plasmid VIBNI_pA	NCBI
ZP_07380973.1<br>Panto	343 aa	26% (Vika)<br>41% (Cre)<br>53% (Dre)	Pantoea sp. aB ctg00071	contig 71 whole genome shotgun sequence	NCBI
ADW76730.1	378 aa	29% (Vika)<br>41% (Cre)<br>42% (Dre)	Rahnella sp. Y9602	plasmid pRAHAQ02	ACLAME
NP_058395.1	393 aa	25% (Vika)<br>27% (Cre)<br>24% (Dre)	Salmonella enterica subsp. enterica serovar Typhi	plasmid R27	ACLAME
ZP_13862083.1<br>Dre	342 aa	24% (Vika)<br>40% (Cre)<br>99.7% (Dre)	Escherichia coli DEC12A	contig.73_1 whole genome shotgun sequence.	NCBI
ZP_16806260.1<br>Dre	342 aa	24% (Vika)<br>40% (Cre)<br>100% (Dre)	Escherichia coli H252	supercont2.12 whole genome shotgun sequence	NCBI
WP_001569361.1<br>Cre	343 aa	28% (Vika)<br>100% (Cre)<br>40% (Dre)	Escherichia coli KTE119	genomic scaffold acEnY-supercont1.4	NCBI
YP_006472.1<br>Cre	343 aa	28% (Vika)<br>99% (Cre)<br>40% (Dre)	Enterobacteria phage P1	Enterobacteria phage P1 complete genome	ACLAME

Supplementary Table 2: Primers used for construction of pEVO, pD and RFP based recombination reporter vectors as well as primers used in pEVO recombination assay. The first column shows the names of primers used in PCR reactions. The vectors that served as recipients of PCR inserts are highlighted in bold in this column. The second column lists the nucleotide sequences of the primers used in the PCR reactions.

Primer name	Nucleotide sequence 5´→ 3´
**Construction of pEVO vectors**	
pEVO-nox fw	GTAGATCTTGAATGTCCTATAATTACACTTATAGGACATTCAGGTCTGACGCTCAGTGGAAC
pEVO-nox rev	GTCTCGAGTGAATGTCCTATAAGTGTAATTATAGGACATTCAGAAATGTGCGGCGGAACCCC
pEVO-pox fw	GTAGATCTGAAACTTTAAATAATAAGTCTTATTTAAAGTTTCGGTCTGACGCTCAGTGGAAC
pEVO-pox rev	GTCTCGAGGAAACTTTAAATAAGACTTATTATTTAAAGTTTCGAAATGTGCGGCGGAACCCC
pEVO-loxP fw	GTAGATCTATAACTTCGTATAGCATACATTATACGAAGTTATTGACTAAATAGGGTCTGACGCTCAGTGGAAC
pEVO-loxP rev	GTCTCGAGGCATAACTTCGTATAATGTATGCTATACGAAGTTATCTATTTAGTCACCGATTTCGGCCTATTGG
pEVO-vox fw	GTAGATCTAATAGGTCTGAGAACGCCCATTCTCAGACGTATTGGTCTGACGCTCAGTGGAAC
pEVO-vox rev	GTCTCGAGGCAATACGTCTGAGAATGGGCGTTCTCAGACCTATTCCGATTTCGGCCTATTGG
pEVO-rox fw	TTGAGATCTTAACTTTAAATAATTGGCATTATTTAAAGTTATCGAACTGTACCGGTTGTTAGTGA
pEVO-rox rev	ATGAGATCTTAACTTTAAATAATGCCAATTATTTAAAGTTAAAGCTTGCATGCCTGCAGATCGAG
pEVO-VloxP fw	GTTAGATCTTCAATTTCCGAGAATGACAGTTCTCAGAAATTGAGGTCTGACGCTCAGTGGAAC
pEVO-VloxP rev	GTTCTCGAGGCTCAATTTCTGAGAACTGTCATTCTCGGAAATTGACCGATTTCGGCCTATTGG
**Construction of pD vectors**	
R6K rev	ACTCTCGAGGAAATGTGCGCGGAACCCC
R6K-nox fw	GTTCTAGACTAGATCTTGAATGTCCTATAATTACACTTATAGGACATTCAGGTCTGACGCTCAGTGGAAC
R6K-pox fw	GTTCTAGACTAGATCTGAAACTTTAAATAATAAGTCTTATTTAAAGTTTCGGTCTGACGCTCAGTGGAAC
R6K-rox fw	ACTTCTAGATAACTTTAAATAATTGGCATTATTTAAAGTTAGGTCTGACGCTCAGTGGAAC
**Construction of RFP based recombination reporter vectors**	
nox-puroR fw	ACCACCGGTTGAATGTCCTATAATTACACTTATAGGACATTCACGCCACCATGACCGAGTACAAGCCC
nox-puroR rev	GATACCGGTTTGAATGTCCTATAAGTGTAATTATAGGACATTCAATTACGCCAAGCTCTAGC
pox-puroR fw	ACCACCGGTGAAACTTTAAATAATAAGTCTTATTTAAAGTTTCCGCCACCATGACCGAGTACAAGCCC
pox-puroR rev	GATACCGGTGAAACTTTAAATAAGACTTATTATTTAAAGTTTCATTACGCCAAGCTCTAGC
loxP-puroR fw	ACCACCGGTATAACTTCGTATAGCATACATTATACGAAGTTATCGCCACCATGACCGAGTACAAGCCC
loxP-puroR rev	GATACCGGTATAACTTCGTATAATGTATGCTATACGAAGTTATATTACGCCAAGCTCTAGC
vox-puroR fw	ACCACCGGTAATAGGTCTGAGAACGCCCATTCTCAGACGTATTCGCCACCATGACCGAGTACAAGCCC
vox-puroR rev	GATACCGGTAATACGTCTGAGAATGGGCGTTCTCAGACCTATTATTACGCCAAGCTCTAGC

rox-puroR fw	ACCACCGGTTAACTTTAAATAATTGGCATTATTTAAAGTTACGCCACCATGA CCGAGTACAAGCCC
rox-puroR rev	GATACCGGTTAACTTTAAATAATGCCAATTATTTAAAGTTAATTACGCCAAG CTCTAGC
FRT-puroR fw	ACCACCGGTGAAGTTCCTATTCTCTAGAAAGAATAGGAACTTCCGCCACCA TGACCGAGTACAAGCCC
FRT-puroR rev	GATACCGGTGAAGTTCCTATTCTTTCTAGAGAATAGGAACTTCATTACGCC AAGCTCTAGC
VloxP-puroR fw	ACCACCGGTTCAATTTCTGAGAACTGTCATTCTCGGAAATTGACGCCACCA TGACCGAGTACAAGCCC
VloxP-puroR rev	GATACCGGTTCAATTTCCGAGAATGACAGTTCTCAGAAATTGAATTACGCC AAGCTCTAGC
**pEVO recombination assay**	
int1	TCTGTTGTTTGTCGGTGAACG
int2	CTTAAACGCCTGGTGCTACG
int3	ATAAATCTGGAGCCGGTGAGC
int4	AAGAGGCATAAATTCCGTCAGC

Supplementary Reference:

Abi-Ghanem, J. *et al.* Engineering of a target site-specific recombinase by a combined evolution- and structure-guided approach. *Nucleic Acids Res* **41**, 2394-2403, doi:10.1093/nar/gks1308 (2013).
